# Amniotic fluid biomarkers predict the severity of congenital cytomegalovirus infection

**DOI:** 10.1172/JCI157415

**Published:** 2022-06-01

**Authors:** Olesya Vorontsov, Lorinne Levitt, Daniele Lilleri, Gilad W. Vainer, Orit Kaplan, Licita Schreiber, Alessia Arossa, Arseno Spinillo, Milena Furione, Or Alfi, Esther Oiknine-Djian, Meital Kupervaser, Yuval Nevo, Sharona Elgavish, Moran Yassour, Maurizio Zavattoni, Tali Bdolah-Abram, Fausto Baldanti, Miriam Geal-Dor, Zichria Zakay-Rones, Nili Yanay, Simcha Yagel, Amos Panet, Dana G. Wolf

**Affiliations:** 1 Clinical Virology Unit, Hadassah-Hebrew University Medical Center and Faculty of Medicine,; 2 Department of Biochemistry, Institute for Medical Research, Israel-Canada (IMRIC), Faculty of Medicine,; 3 Lautenberg Center for General and Tumor Immunology, Faculty of Medicine, and; 4 Department of Obstetrics and Gynecology, Hadassah-Hebrew University Medical Center and Faculty of Medicine, The Hebrew University of Jerusalem, Jerusalem, Israel.; 5 Department of Microbiology and Virology, Istituto di Ricovero e Cura a Carattere Scientifico (IRCCS) Policlinico San Matteo Foundation and University of Pavia, Pavia, Italy.; 6 Department of Pathology, Hadassah-Hebrew University Medical Center and Faculty of Medicine, The Hebrew University of Jerusalem, Jerusalem, Israel.; 7 Maccabi Healthcare Services, Central Laboratory, Rehovot, Israel.; 8 Department of Obstetrics and Gynecology, IRCCS Policlinico San Matteo Foundation and University of Pavia, Pavia, Italy.; 9 The De Botton Protein Profiling Institute of the Nancy and Stephen Grand Israel National Center for Personalized Medicine, Weizmann Institute of Science, Rehovot, Israel.; 10 Info-CORE, Bioinformatics Unit of the I-CORE,; 11 School of Computer Science and Engineering,; 12 Department of Microbiology and Molecular Genetics, IMRIC, Faculty of Medicine, and; 13 Faculty of Medicine, The Hebrew University of Jerusalem, Jerusalem, Israel.; 14 Department of Speech and Hearing, Hadassah-Hebrew University Medical Center, Jerusalem, Israel.

**Keywords:** Infectious disease, Virology, Diagnostics, Obstetrics/gynecology, Proteomics

## Abstract

**BACKGROUND:**

Cytomegalovirus (CMV) is the most common intrauterine infection, leading to infant brain damage. Prognostic assessment of CMV-infected fetuses has remained an ongoing challenge in prenatal care, in the absence of established prenatal biomarkers of congenital CMV (cCMV) infection severity. We aimed to identify prognostic biomarkers of cCMV-related fetal brain injury.

**METHODS:**

We performed global proteome analysis of mid-gestation amniotic fluid samples, comparing amniotic fluid of fetuses with severe cCMV with that of asymptomatic CMV-infected fetuses. The levels of selected differentially excreted proteins were further determined by specific immunoassays.

**RESULTS:**

Using unbiased proteome analysis in a discovery cohort, we identified amniotic fluid proteins related to inflammation and neurological disease pathways, which demonstrated distinct abundance in fetuses with severe cCMV. Amniotic fluid levels of 2 of these proteins — the immunomodulatory proteins retinoic acid receptor responder 2 (chemerin) and galectin-3–binding protein (Gal-3BP) — were highly predictive of the severity of cCMV in an independent validation cohort, differentiating between fetuses with severe (*n =* 17) and asymptomatic (*n =* 26) cCMV, with 100%–93.8% positive predictive value, and 92.9%–92.6% negative predictive value (for chemerin and Gal-3BP, respectively).

**CONCLUSION:**

Analysis of chemerin and Gal-3BP levels in mid-gestation amniotic fluids could be used in the clinical setting to profoundly improve the prognostic assessment of CMV-infected fetuses.

**FUNDING:**

Israel Science Foundation (530/18 and IPMP 3432/19); Research Fund – Hadassah Medical Organization.

## Introduction

Congenital cytomegalovirus (cCMV) is the most common intrauterine infection, occurring in an average of 0.64% live births worldwide and leading to infant brain damage, neurodevelopmental disabilities, and sensorineural hearing loss (1). Approximately 20% of congenitally infected infants have neurological and/or audiological disabilities, which can be apparent at birth or develop later during childhood (1, 2). cCMV may follow maternal primary and nonprimary infection, and the risk of severe neurological deficits is greatest when maternal infection is acquired during the first trimester (1, 2). Despite the public health burden of cCMV, systematic screening of pregnant women for CMV infection is not officially performed, mainly due to the lack of established prenatal antiviral treatments and early prenatal biomarkers of fetal/neonatal disease. However, improvements in the diagnosis of primary maternal infection (with utilization of IgG avidity assays) and in the prenatal diagnosis of fetal infection by CMV real-time quantitative PCR (RT-qPCR) in amniotic fluid have triggered extensive de facto screening of pregnant women for CMV infection in European countries and Israel (3–5). Recent treatment trials in pregnant women, using high-dose oral valacyclovir and high-dose, CMV-specific hyperimmune globulins, have indicated the effectiveness of timely antiviral treatment in reducing intrauterine transmission and improving outcomes for infants (2–7). Together, these diagnostic and therapeutic developments underscore the need for early prognostic biomarkers of cCMV severity.

Currently, antenatal assessment of cCMV severity is mostly based on fetal imaging (by targeted ultrasound and MRI), which is compromised by suboptimal predictive values until late in the third trimester (3, 8). Earlier viral and nonviral parameters in amniotic fluid and fetal blood have been examined for their ability to predict the severity of fetal infection. However, a high viral DNA load is a common finding in CMV-infected amniotic fluids and is not predictive of fetal disease, and the analysis of fetal blood parameters (platelet counts, IgM levels, CMV DNA levels, and β-2-microglobulin levels) requires an invasive cordocentesis procedure and is not routinely performed (1, 3, 8, 9). In 2 studies, an amniotic fluid 34-peptide classifier identified by peptidome analysis and HLA-G levels have been shown to predict the severity of fetal infection (each in a small number of symptomatic/asymptomatic fetuses), yet none has been introduced into clinical use (10, 11).

The search for biomarkers that would allow prognostic assessment of cCMV-infected fetuses (desirably when the diagnosis of fetal infection is made) is ongoing. Toward this goal, we have used global proteome analysis of mid-gestation amniotic fluid samples of fetuses with classified cCMV infection severity. We identified and further validated highly predictive amniotic fluid protein biomarkers for the severity of cCMV.

## Results

### Identification of amniotic fluid proteins associated with the severity of cCMV.

**Figure 1:**
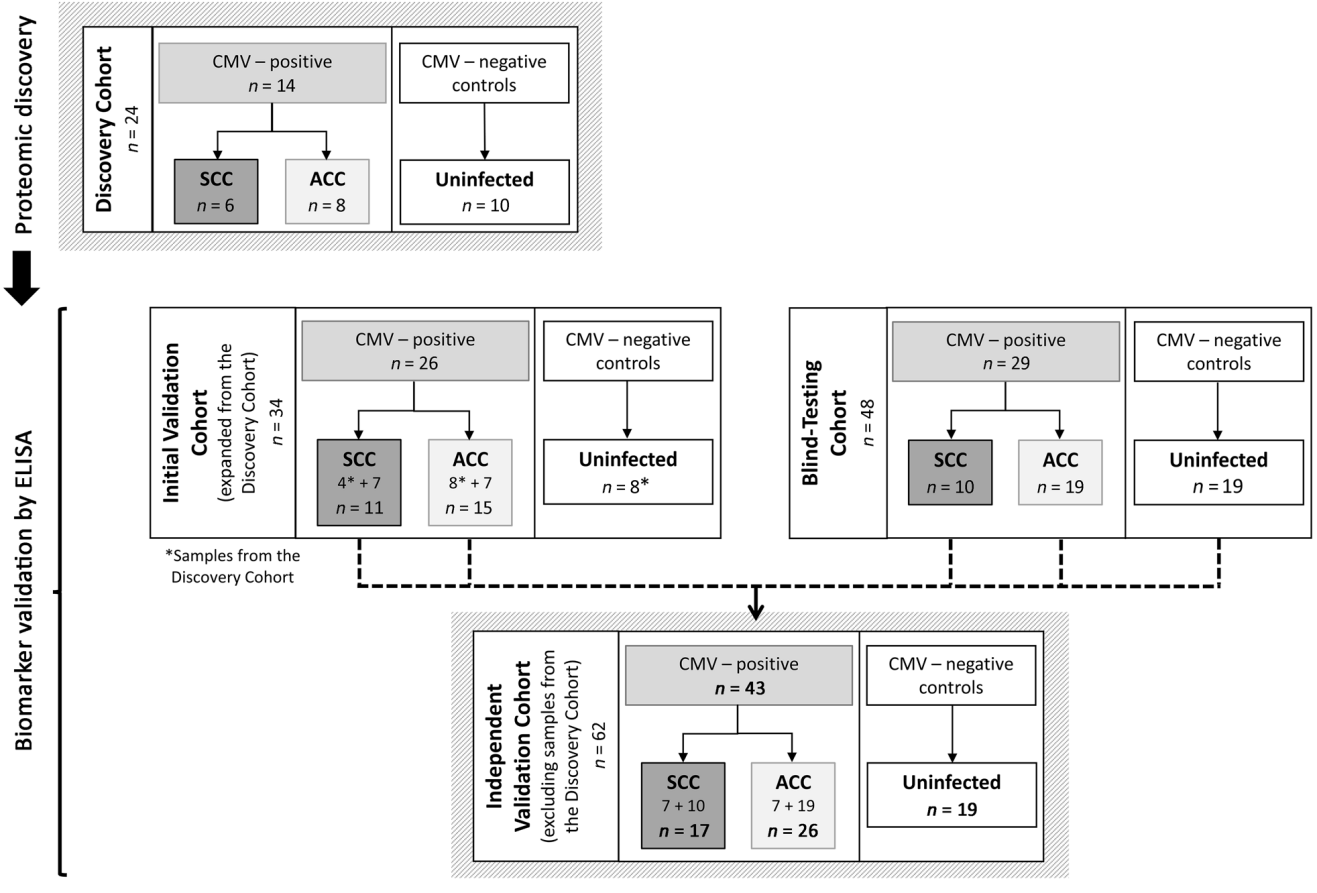
Schematic presentation of study outline and patient cohorts. SCC, severe cCMV infection; ACC, asymptomatic cCMV infection.

**Figure 2:**
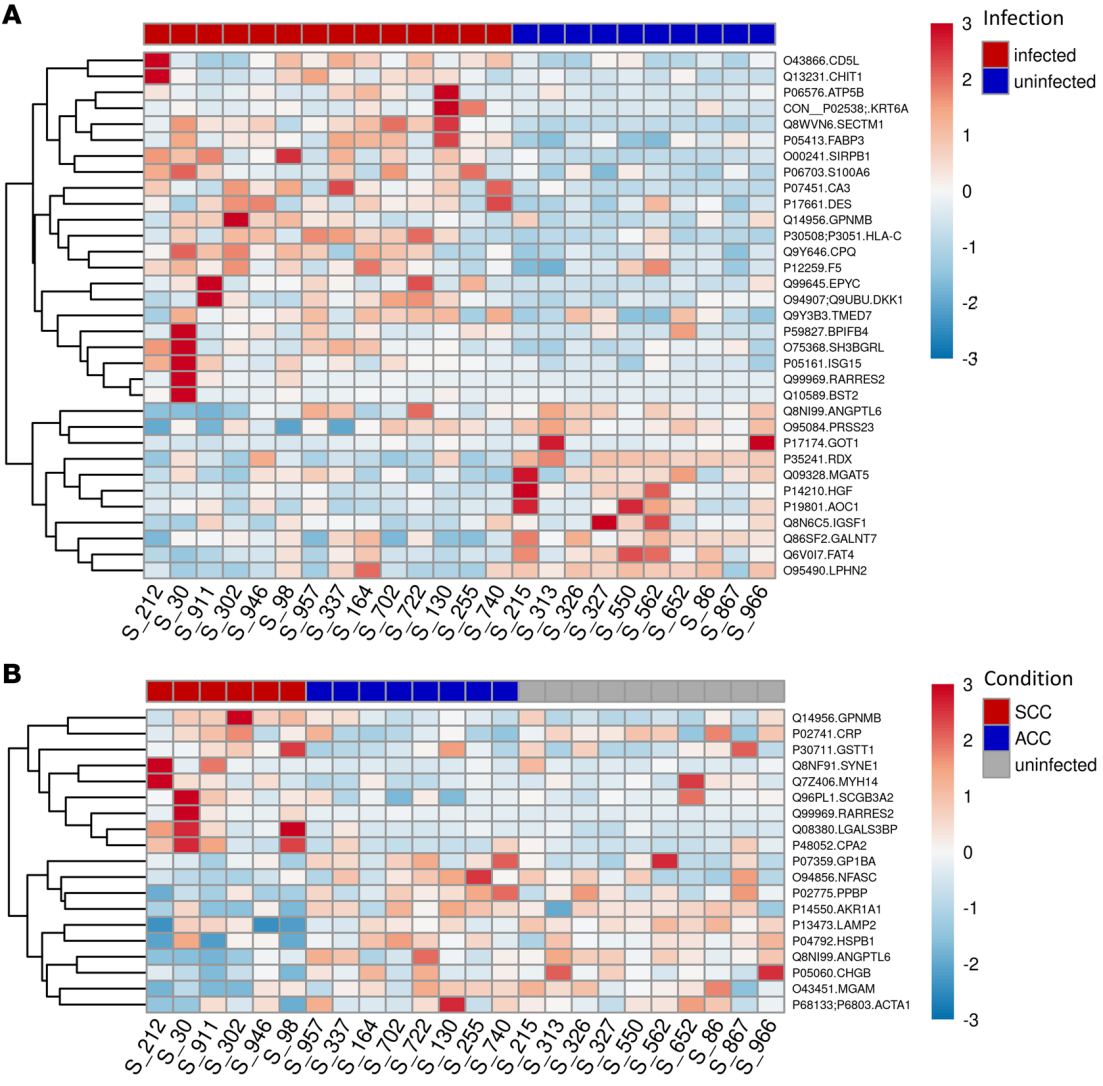
Heatmaps showing differential excretion of amniotic fluid proteins between CMV-infected and uninfected fetuses and between fetuses with severe and asymptomatic cCMV. Heatmaps were drawn using normalized LFQ intensity values of the differentially excreted (>2-fold) proteins (indicated by symbols and accession numbers) after scaling per protein (rows) over all drawn samples (columns). (**A**) Differential excretion of amniotic fluid proteins between CMV-infected and uninfected fetuses. (**B**) Differential excretion of amniotic fluid proteins between fetuses with severe cCMV and those with asymptomatic cCMV. In **B**, the relative signals of the proteins that were differentially excreted between fetuses with severe cCMV and those with asymptomatic cCMV are also shown for the uninfected fetuses. S_number, sample number; RARRES2, retinoic acid receptor responder 2 (chemerin); LGALS3BP, galectin-3–binding protein (Gal-3BP).

With the aim of an unbiased identification of amniotic fluid proteins associated with cCMV severity, we analyzed the amniotic fluid proteome of 6 fetuses with severe cCMV and 8 fetuses with asymptomatic cCMV (the latter were found to be asymptomatic neonates at birth and remained asymptomatic for at least 24 months after birth) and 10 CMV-negative (uninfected) fetuses (Figure 1 and Supplemental Table 1; supplemental material available online with this article; https://doi.org/10.1172/JCI157415DS1). In total, 1174 human-origin proteins were identified and quantified in the amniotic fluid samples. We obtained qualitative and quantitative data for each identified protein, along with the relevant relative intensity comparisons between samples. The fold change for each comparison (i.e., infected versus uninfected; severe versus asymptomatic cCMV) was calculated on the basis of protein label-free quantification (LFQ) intensity. We identified 59 proteins that were significantly differentially excreted between infected and uninfected fetuses (Supplemental Table 2) and 29 proteins that were significantly differentially excreted between the symptomatic and asymptomatic infected fetuses (see Table 1 for the list of proteins differentially excreted by more than 2-fold and their basic characteristics and Supplemental Table 3 for the complete list of differentially excreted proteins). To better delineate the distribution of the differentially excreted proteins among the compared groups and individual fetuses, their normalized LFQ intensity signals were visualized in heatmaps. Figure 2 shows the case-to-case variability, yet clearly demonstrates the distinct protein patterns distinguishing between infected and uninfected fetuses (Figure 2A) and between fetuses with severe and asymptomatic cCMV (Figure 2B).

### Ingenuity Pathway Analysis of the differentially excreted proteins.

**Figure 3:**
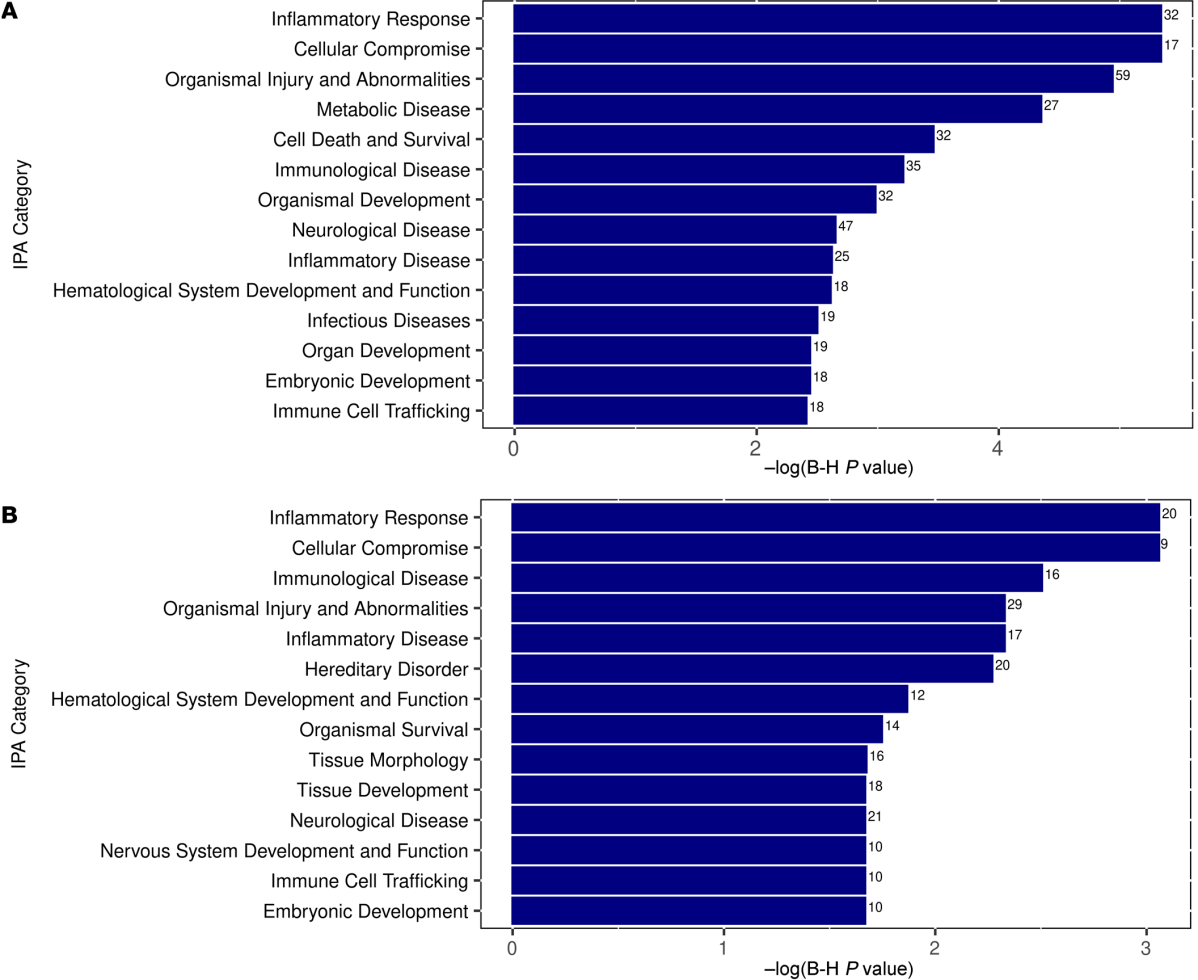
Plots of the top enriched pathways of differentially excreted proteins between CMV-infected and uninfected fetuses and between fetuses with severe and asymptomatic cCMV, based on IPA. (**A**) Top enriched pathways of differentially excreted proteins between CMV-infected and uninfected fetuses. (**B**) Top enriched pathways of differentially excreted proteins between fetuses with severe cCMV and fetuses with asymptomatic cCMV. The numbers adjacent to each category in the plots indicate the number of differentially excreted proteins related to each of the shown pathways.

To gain insight into the biological pathways and predicted functions of the identified differentially excreted amniotic fluid proteins, we performed Ingenuity Pathway Analysis (IPA) (QIAGEN). Focusing on pathways related to diseases and biofunctions, the top relevant categories enriched in infected versus uninfected fetuses included inflammatory response, cellular compromise, and organismal injury and abnormalities (see Figure 3A for additional enriched pathways). These findings reflected pathways enriched by both severe and asymptomatic infection. Interestingly, when we specifically compared between the severe and asymptomatic cCMV infection groups, the top enriched categories included inflammatory response, cellular compromise, immunological disease, and organismal injury and abnormalities (Figure 3B). Also among the enriched categories were neurological disease and nervous system development and function. These findings could be linked to the dominant neurological abnormalities of cCMV and could further implicate the immune/inflammatory-mediated pathogenesis of severe cCMV.

### Amniotic fluid retinoic acid receptor responder 2 and galectin-3–binding protein levels distinguish severe from asymptomatic cCMV.

Having identified a group of proteins differentially excreted in infected fetuses with severe cCMV, we selected 2 of the identified proteins for validation using ELISA measurement: retinoic acid receptor responder 2 (chemerin), a chemoattractant protein with regulatory roles in immune and metabolic processes, and galectin-3–binding protein (Gal-3BP), a multifunctional immunomodulating glycoprotein (Table 1). These 2 candidate biomarkers stood out and were thus chosen for further analysis, given their significant and consistent relative abundance in the symptomatic group (Table 1 and Figure 2B), their involvement in the enriched disease-related pathways (as revealed by IPA), and the availability of specific quantitative ELISAs (reportedly used in clinical body fluids).

We evaluated the ability of chemerin and Gal-3BP levels to separate between fetuses with severe and asymptomatic cCMV in (a) an initial validation cohort (extended from the discovery cohort), aimed to confirm the differential excretion of the proteins by ELISAs, and (b) a blind (outcome-blinded) testing cohort from 2 unrelated centers (Figure 1 and Supplemental Table 2). Gestational age at amniocentesis was 20–23 weeks (except for 1 symptomatic fetus, who underwent amniocentesis at week 27; case 758 in Supplemental Table 4). The mean gestational age at amniocentesis was 21 weeks for fetuses with severe and asymptomatic cCMV and uninfected fetuses. In the case of all fetuses with severe cCMV except 1, pregnancies were terminated because of findings of severe defects by prenatal imaging (Supplemental Table 1 and Supplemental Table 4). Nine of the fetuses underwent an autopsy, which confirmed the severe fetal pathology. Gestational age at the appearance of severe cCMV imaging findings ranged from 16–31 weeks. In 7 fetuses (cases 30 and 911 in Supplemental Table 1, and cases 333, 254, 486, IT23, and IT29 in Supplemental Table 4), severe cCMV findings on imaging appeared for the first time at the gestational age of 25–31 weeks, 4–9 weeks after the time of the amniocentesis. For fetuses that were asymptomatic, the median postnatal follow-up assessment was done at 36 months (range 12–68 months).

**Figure 4:**
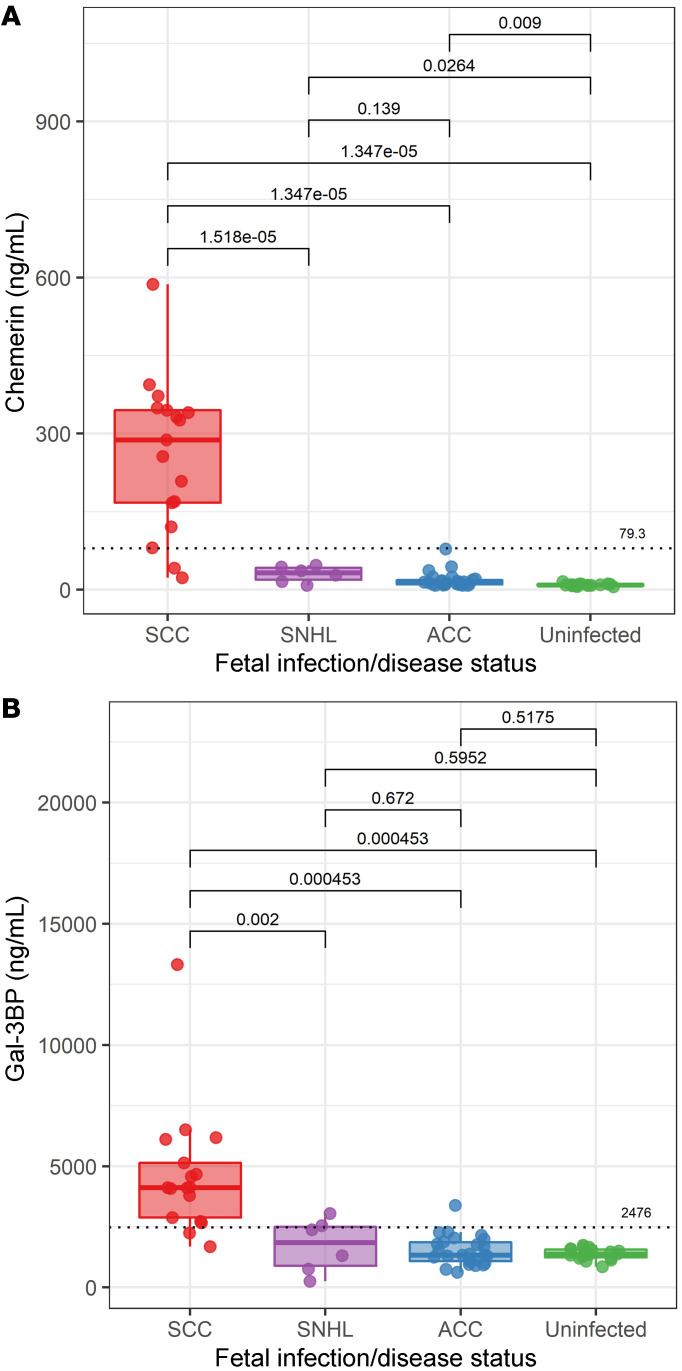
Amniotic fluid chemerin and Gal-3BP levels in fetuses with severe cCMV, SNHL, or asymptomatic cCMV, and in uninfected fetuses. The levels shown were analyzed in an independent validation cohort of 17 fetuses with severe cCMV, 26 fetuses with asymptomatic cCMV, and 19 control uninfected fetuses, as well as in 6 neonates with isolated SNHL. Chemerin (**A**) and Gal-3BP (**B**) levels in amniotic fluid according to infection/disease status. In the plots, the dotted horizontal lines represent optimal predictive cutoff values between severe cCMV and asymptomatic cCMV, derived by ROC analysis. The figures were generated using the R ggplot2 package (version 3.1.0). Box plots were generated using geom_boxplot default parameters, such that the box represents the 25th–75th percentiles and the whiskers from the box to the largest or smallest value are no more than 1.5 times the IQR from the box (the IQR is the distance between the first and third quartiles). *P* values were calculated by Bonferroni’s correction for multiple comparisons.

The results clearly demonstrated the significantly higher levels of chemerin and Gal-3BP in fetuses with severe cCMV compared with those with asymptomatic cCMV (Figure 4; see also Supplemental Figure 1, A and B, and Supplemental Figure 2, A and B, for analysis of the subcohorts). Of note, chemerin levels were also mildly elevated in infected, asymptomatic fetuses compared with levels in uninfected fetuses, suggesting its initial triggering by infection per se. The high accuracy of both chemerin and Gal-3BP in differentiating between fetuses with severe cCMV and those with asymptomatic cCMV was first shown by receiver operating characteristic (ROC) analysis in the initial validation cohort, which also served to define primary threshold values for the 2 proteins, that would discriminate between severe and asymptomatic cCMV (Supplemental Table 5). Strikingly, the defined cutoff levels of each of the 2 proteins predicted the severity of cCMV in the blind-testing cohort, with 90% sensitivity, 100% specificity, 100% positive predictive value (PPV), and 95% negative predictive value (NPV) (Supplemental Table 5). It should be noted that in the 1 severe cCMV case, which was wrongly predicted (by the biomarkers) to be asymptomatic (case IT23 in Supplemental Table 4), severe cCMV imaging findings appeared for the first time at gestational week 28, seven weeks after the amniocentesis. Additionally, 1 fetus in the blind-testing cohort (case IT29 in Supplemental Table 4), who was classified as having severe cCMV (based on the biomarkers’ levels), was categorized as having severe cCMV based on the presence of severe cerebral abnormalities on MRI and on fetal blood parameters (measured at the Italian center; ref. 9). The child was subsequently diagnosed as symptomatic at birth, received 6 months of oral valganciclovir therapy, and at 12 months of age his developmental and hearing examinations were normal.

In an overall analysis of an independent validation cohort (including cases unrelated to the discovery cohort; Figure 1), chemerin and Gal-3BP levels showed 88.2% sensitivity (each), 100%–96.2% specificity, 100%–93.8% PPV, and 92.9%–92.6% NPV, with a 0.98–0.97 AUC (for chemerin and Gal-3BP, respectively), in differentiating 17 fetuses with severe cCMV from 26 fetuses with asymptomatic cCMV (Table 2). By comparison, amniotic fluid viral DNA levels, although significantly higher in symptomatic compared with asymptomatic fetuses (6.50 ± 0.60 vs. 5.17 ± 1.10 log DNA copies/mL; *P <* 0.001), demonstrated an inferior prognostic performance with much lower specificity and PPV (Table 2). Moreover, in 6 fetuses, high levels of chemerin and Gal-3BP preceded the appearance of severe brain lesions by 4–9 weeks (cases 30 and 911 in Supplemental Table 1, and cases 333, 254, 486, and IT29 in Supplemental Table 4).

Having shown that chemerin and Gal-3BP accurately distinguish severe from asymptomatic cCMV, we further examined their levels in amniotic fluid specimens from 6 infants with isolated cCMV-related sensorineural hearing loss (SNHL). SNHL (moderate to profound) was diagnosed at birth in 2 of the infants and developed later (at 6–24 months of age) in 4 infants. Interestingly, the levels of chemerin and Gal-3BP in this group were significantly lower than those detected in infants with severe cCMV (Figure 4). While the median levels of chemerin and Gal-3BP in infants with SNHL were somewhat higher than those in the asymptomatic infants (chemerin 32.3 vs. 13.5 ng/mL; Gal-3BP 1846 vs. 1341 ng/mL), the differences were not statistically significant (Figure 4). Given the small number of infants in the SNHL group, it would be important to investigate the predictive values of the biomarkers in a larger cohort of infants with isolated hearing deficits. Together, these data reveal distinct amniotic fluid protein patterns in amniotic fluid of fetuses with severe cCMV compared with that of fetuses with asymptomatic cCMV and identify chemerin and Gal-3BP as highly predictive biomarkers of cCMV severity.

## Discussion

The identification of effective antenatal biomarkers of cCMV infection severity has been hampered by the limited biological understanding of the pathogenesis of cCMV disease and by the small numbers of affected pregnancies with known clinical outcomes. Addressing this gap, we report here on the identification and clinical validation of the immune-modulating proteins chemerin and Gal-3BP as what we believe to be new biomarkers that can be quantified in mid-gestation amniotic fluid samples to assess the prognosis of CMV-infected fetuses.

By performing unbiased proteome analysis of the amniotic fluid of fetuses with severe or asymptomatic cCMV and of uninfected fetuses (the discovery cohort), we identified proteins that were differentially excreted between CMV-infected and uninfected fetuses and further identified 29 proteins that showed a significant (*P* ≤ 0.05) differential abundance between fetuses with severe cCMV and those with asymptomatic cCMV. Interestingly, pathway analysis of the proteins showing distinct abundance in fetuses with severe cCMV revealed that the top enriched pathways were for the inflammatory response, cellular compromise, and immunological disease, along with pathways for organismal injury and abnormalities and neurological disease, suggesting a connection between aberrant inflammation at the maternal-fetal interface and the development of cCMV-related fetal brain damage. These findings, linked to cCMV severity, add new aspects to previous studies that showed proinflammatory cytokine patterns in CMV-infected amniotic fluid and placental tissues (with no fetal disease data included in the report), and that demonstrated a correlation between the extent of placental and fetal brain immune cell infiltration and the severity of cCMV (12–16). Together, the combined findings argue for the immunopathogenesis of cCMV-associated fetal damage.

Toward the goal of identifying prognostic biomarkers of cCMV severity, we selected chemerin and Gal-3BP as primary candidates for validation on the basis of their consistently increased abundance in symptomatic fetuses (Table 1 and Figure 2B) and their involvement in the enriched immune damage–related pathways (as revealed by IPA). Using quantitative ELISAs, we confirmed the significantly (*P* < 0.001) elevated levels of chemerin and Gal-3BP in amniotic fluid of fetuses with severe cCMV compared with that of fetuses with asymptomatic cCMV (Figure 4, Supplemental Figure 1, and Supplemental Figure 2) and further defined outcome-differentiating cutoff values for both proteins. Importantly, we demonstrated the high predictive accuracy of these 2 identified biomarkers in distinguishing 17 fetuses with severe cCMV from 26 fetuses with asymptomatic cCMV (included in an independent validation cohort from 3 unrelated centers), showing 88.2% sensitivity (for each), 100%–96.2% specificity, 100%–93.8% PPV, and 92.9%–92.6% NPV, with 0.98–0.97 AUC (for chemerin and Gal-3BP, respectively). By comparison, and in accordance with previous reports, amniotic fluid viral load levels had considerably lower predictive values (Table 2 and refs. 1–3). Notably, the presence of high levels of chemerin and Gal-3BP in the amniotic fluid preceded the appearance of severe cCMV imaging findings by 4–9 weeks in 6 fetuses, further highlighting the potential prognostic value of these proteins. Further analysis of a small group of infants with isolated SNHL showed significantly (*P* < 0.01) lower chemerin and Gal-3BP levels compared with infants with severe cCMV (Figure 4), yet studies of this potentially heterogenous group should be expanded.

To the best of our knowledge, chemerin and Gal-3BP have not been previously explored in the context of CMV infection. Chemerin is a chemoattractant adipokine (belonging to a group of adipose-secreted proteins) implicated in the regulation of inflammatory conditions (including inflammatory nervous system conditions) and metabolic processes (Table 1 and refs. 17–21). Increased maternal serum chemerin levels have been associated with pregnancy complications, such as gestational diabetes mellitus and preeclampsia (22, 23). It is tempting to speculate that elevated chemerin levels at the maternal-fetal interface in severe cCMV cases could be mechanistically linked to the excessive placental leukocyte infiltration that is characteristic of severe cCMV pathology. Gal-3BP is an immunomodulatory glycoprotein studied in the context of neoplastic transformation and cancer progression (24). Gal-3BP has been shown to be induced by various viral infections, including HIV, HBV, hepatitis C virus (HCV), hantavirus, and dengue virus infections, and has been studied as a serologic biomarker of HBV-related hepatocellular carcinoma and HIV progression (25, 26). Our findings warrant mechanistic studies of the role of these multifunctional innate immune effectors in cCMV-related brain damage. They could further trigger studies of the potential role of the identified biomarkers in the neurodevelopmental abnormalities associated with other major congenital infections (such as Zika virus infections).

Analysis of chemerin and Gal-3BP in mid-gestation amniotic fluids of infected fetuses (by accessible ELISAs) could be used in the clinical setting to substantially improve the prognostic assessment of infected fetuses and to identify a window of opportunity for potential therapeutic interventions.

There are several limitations in this study. First, the inclusion of amniotic fluid samples linked to strictly defined cCMV severity limited the sample size. However, the discovery cohort proved to be sufficient for the identification of a group of differentially excreted proteins in fetuses with severe cCMV. We were further able to validate the selected biomarkers in a clinical cohort of a relatively considerable size (given the inherent size limitation in all cCMV biomarker studies, dictated by the fact that many of the cases of infected fetuses lack clear outcome data) from unrelated centers, which yielded substantial findings and supported the generalizability of our results. Second, both the discovery and validation cohorts in this study were confined to severe and asymptomatic cCMV cases and did not reflect the spectrum of fetuses with mild-to-moderate symptomatic cCMV, presuming a mixed and less-defined phenotype in these cases. Thus, the proportion of fetuses with severe cCMV in this study cohort was much higher than their real prevalence among cCMV cases, a difference that may affect the eventual cutoff levels and predictive value of the identified biomarkers. Nevertheless, we believe that the marked dichotomy of the groups, avoiding the heterogeneity of intermediate disease phenotypes, facilitated the discovery and validation of differentiating biomarkers. Future prospective studies are needed to assess the predictive values of these biomarkers in larger cohorts, including infants with cCMV-related hearing deficits. While we focused our attention on chemerin and Gal-3BP, additional candidate proteins (shown in Table 1 and Figure 2A) may be of interest for future studies, as we believe that more biomarkers would be needed to address the complexity of cCMV outcomes.

In summary, the data presented here identify the immunomodulatory proteins chemerin and Gal-3BP as highly predictive amniotic fluid biomarkers of cCMV infection severity, which could guide early prognostic stratification and potentially personalized treatment of cCMV-infected fetuses. Our findings provide insights for further mechanistic studies of inflammatory pathways and treatable targets involved in the progression of CMV-related fetal brain damage.

## Methods

### Study design and patient population.

Amniotic fluid samples, obtained at 20–23 weeks of gestation (and at least 6 weeks after the assumed infection) from women diagnosed with primary CMV infection (as part of the routine diagnosis of fetal infection) were retrieved for retrospective analysis. Samples were stored at –80°C until the analysis. Included in the study were amniotic fluid samples of CMV-infected fetuses with characterized cCMV severity, classified as either severe or asymptomatic cCMV infection (see *Definition of severe and asymptomatic infection* below). The classification was based on review of the antenatal data, including fetal imaging, and pathological examination following termination of pregnancy (TOP), and of the neonatal/postnatal follow-up data. Contemporaneous CMV-negative (uninfected) amniotic fluid samples, obtained under the same indications and conditions were analyzed in parallel. The study involved a proteomics discovery phase, carried out in a discovery cohort (composed of samples retrieved from the Hadassah Medical Center Clinical Virology Laboratory), and a biomarker validation phase. The biomarker validation phase was performed in (a) an initial validation cohort, which included samples from the discovery cohort (subjected to the proteome analysis) and additional independent samples (retrieved from the Hadassah Medical Center Clinical Virology Laboratory), and (b) an outcome-blinded (blind-testing) cohort from the Fondazione IRCCS Policlinico San Matteo Virology Laboratory in Pavia, Italy, and the Maccabi Healthcare Services Central Laboratory, in Rehovot, Israel (Figure 1; see also the Supplemental Methods for a detailed cohort description). Together, the 62 independent cases (which were not included in the discovery cohort: *n* = 17 fetuses with severe cCMV; *n* = 26 fetuses with asymptomatic cCMV; *n* = 19 uninfected fetuses) constituted an independent validation cohort (Figure 1).

### Definition of severe and asymptomatic cCMV infection.

Fetuses were classified as severely affected when severe cerebral anomalies were identified by prenatal ultrasound and/or MRI as previously defined (e.g., head circumference <2 SDs of normal, ventriculomegaly, white matter abnormalities and cavitations, intracerebral hemorrhage, delayed cortical development; ref. 27). After the diagnosis of fetal infection by amniocentesis, fetal scans were repeated monthly until delivery or TOP. Fetal brain MRI was performed at 30–32 weeks’ gestation. All scan reports were reviewed again by 2 independent experts for the purpose of inclusion in the present study. After delivery, all newborns were evaluated for cCMV disease status by blood tests, audiological and ophthalmologic tests, and cerebral ultrasound imaging. Follow-up included periodic cognitive, developmental, and auditory evaluation. The cases were classified in the study as asymptomatic cCMV infection when all clinical and laboratory parameters were normal at birth and upon postnatal follow-up of at least 12 months. Fetuses from terminated pregnancies that had normal or mildly abnormal imaging findings were excluded from the study because of insufficient outcome data.

### CMV DNA load analysis.

The viral DNA load in amniotic fluid was determined by RT-qPCR as previously described (see Supplemental Methods; ref. 28).

### Sample preparation, liquid chromatography–mass spectrometry, and data processing.

Amniotic fluid (150 μL) was loaded onto a serum depletion column, followed by an in-solution tryptic digestion and a desalting step. The resulting peptides were analyzed using nanoflow liquid chromatography (nanoAcquity, Waters) coupled to high-resolution, high mass-accuracy mass spectrometry (Q Exactive HFX, Thermo Fisher Scientific). See Supplemental Methods for a detailed description. The LFQ intensities were extracted and used for further calculations with Perseus, version 1.6.0.7. Decoy hits were filtered out, as well as proteins that were identified on the basis of a modified peptide only, and gene ontology (GO) annotations were added. The LFQ intensities were log transformed, and only proteins that had at least 3 valid values in at least 1 experimental group were kept. The remaining missing values were imputed. A *t* test was performed to identify significant differential protein expression between the groups, and a *P* value of 0.05 or less was considered statistically significant. The definition of differential protein expression also required a minimum fold change of greater than 1.5 between the groups. The mass spectrometry proteomics data have been deposited with the ProteomeXchange Consortium via the PRIDE (Proteomics Identifications Database) partner repository with the data set identifier PXD029105 (http://www.ebi.ac.uk/pride/archive/projects/PXD029105).

### IPA of differentially excreted proteins.

Pathway and molecular function and disease enrichment analysis of the significantly differentially excreted proteins was carried out using IPA (QIAGEN, https://www.qiagenbioinformatics.com/products/ingenuity-pathway-analysis). Plots of selected IPA categories (based on IPA for B-H *P* values) were generated using the ggplot2 R graphical package (https://ggplot2.tidyverse.org).

### Chemerin and Gal-3BP immunoassays.

Amniotic fluid chemerin and Gal-3BP protein levels were determined using commercially available quantitative sandwich enzyme immunoassays (Quantikine ELISA, R&D Systems, catalogs DCHM00 and DGBP30B for chemerin and Gal-3BP, respectively), according to the manufacturer’s instructions. Serial dilutions of amniotic fluid samples were initially tested to determine the dilution required to maintain the linearity of the assay within the expected dynamic range. All amniotic fluid samples were tested in duplicate. Four samples (2 CMV-negative and 2 CMV-positive) were included in each run, along with the appropriate calibrators and controls provided by the manufacturer, to monitor potential variability among assays. Protein concentrations were interpolated from the calibration curve using a 4-parameter logistic curve fit.

### Statistics.

Statistical analysis was performed using SPSS, version 26.0 (IBM). Comparisons of chemerin and Gal-3BP expression levels and CMV DNA loads between amniotic fluid samples of fetuses with severe cCMV, fetuses with asymptomatic CMV infection, and CMV-negative fetuses were analyzed using a 2-tailed *t* test or the nonparametric Mann-Whitney *U* test (the latter was used for non-normally distributed variables) for quantitative continuous variables. The identification of an intersection point with high specificity and sensitivity in order to distinguish between fetuses with severe and those with asymptomatic infection based on chemerin, Gal-3BP, or viral DNA levels (cutoff values) was performed using ROC analysis. All tests applied were 2 tailed, and a *P* value of 0.05 or less was considered statistically significant. To account for multiple comparisons, Bonferroni’s post hoc test was applied for correction of the significance level. 

### Study approval.

This study was approved by the IRBs of Hadassah-Hebrew University Medical Center (0273-18-HMO), Maccabi Healthcare Services (ASMC 0069), and the Fondazione IRCCS Policlinico San Matteo (P-20100035854 and P-​20180075214), with a waiver of informed consent.

## Author contributions

DGW, LL, SY, and AP designed the studies. OV, OK, MK, DL, LS, OA, and EOD conducted experiments. OV, LL, DL, LS, AA, AS, MF, MZ, FB, MGD, and NY acquired the data. OV, GWV, OK, MK, YN, SE, MY, TBA, ZZR, AP, and DGW analyzed the data. DGW, OV, and LL wrote the manuscript. Assignment of the authorship order for the 2 co–first authors was based on their relative scientific contributions.

## Supplementary Material

Supplemental data

ICMJE disclosure forms

## Figures and Tables

**Table 1:**
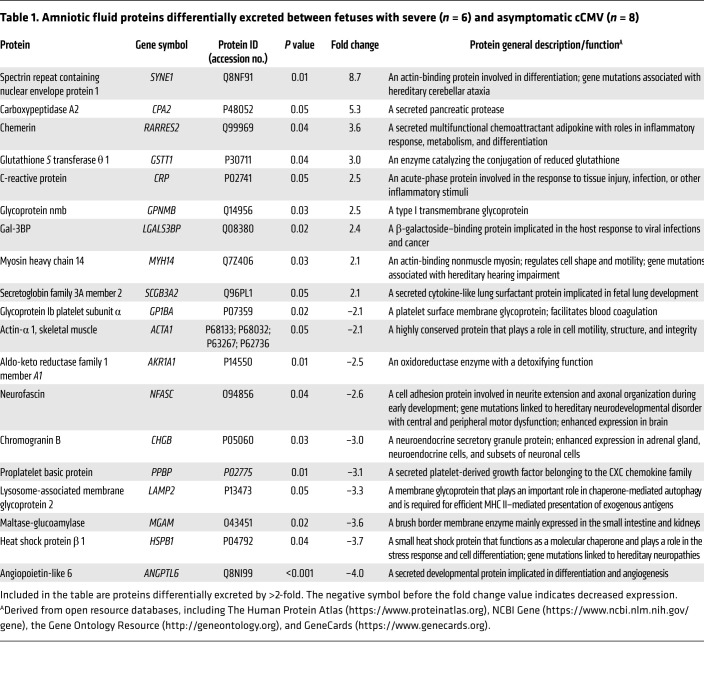
Amniotic fluid proteins differentially excreted between fetuses with severe (*n* = 6) and asymptomatic cCMV (*n* = 8)

**Table 2:**
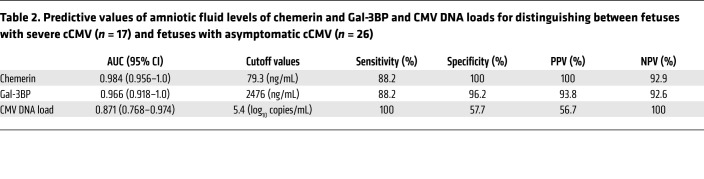
Predictive values of amniotic fluid levels of chemerin and Gal-3BP and CMV DNA loads for distinguishing between fetuses with severe cCMV (*n* = 17) and fetuses with asymptomatic cCMV (*n* = 26)

## Version 1 04/19/2022

In-Press Preview

## Version 2 06/01/2022

Electronic publication
