# Supplementary material for: Amniotic fluid biomarkers predict the severity of congenital cytomegalovirus infection
Source: J Clin Invest. 2022 Jun 1;132(11):e157415. doi: 10.1172/JCI157415 (PMC9151692; doi:10.1172/JCI157415)
Supplement: Supplemental data [file jci-132-157415-s188.pdf]

SUPPLEMENTAL MATERIAL

Methods

Patient cohorts

Proteome analysis was carried out in a Discovery cohort, which included amniotic fluid samples of six fetuses with severe cCMV and eight asymptomatic CMV-infected fetuses, and ten uninfected fetuses, retrieved from the databases of the Hadassah Medical Center Clinical Virology Laboratory (Hadassah lab) (Figure 1, Supplementary Table 1). Validation of selected biomarkers (by specific immunoassays) was carried out in two stages: 1) Using an Initial Validation cohort of 11 fetuses with severe cCMV and 15 asymptomatic CMV-infected fetuses, and eight uninfected fetuses; This cohort, aimed to confirm the proteomics finding by ELISA and to define the proteins' primary cutoff levels for predictive assessment, included available samples (for which sufficient amount of material was available) from the Discovery cohort and additional unrelated samples retrieved from the Hadassah lab (Figure 1, Supplementary Table 1, and Supplementary Table 4). 2) An unrelated clinically-blinded (Blind)-Testing cohort of ten fetuses with severe cCMV and 19 asymptomatic CMV-infected fetuses, and 19 uninfected fetuses from the Fondazione IRCCS Policlinico San Matteo Virology Laboratory, Pavia, Italy (San Matteo lab), and the Maccabi Healthcare Services Central Laboratory, Rehovot, Israel (Maccabi lab) (Supplementary Table 4). Together, the 62 samples not included in the Discovery cohort (17 severe cCMV; 26 asymptomatic cCMV; 19 uninfected) constituted an Independent Validation cohort (Figure 1, and Supplementary Table 4). The predicted classification of this cohort (severe versus asymptomatic), based on the detected biomarker levels, were first blindly defined and submitted by the Hadassah lab investigators, and the biomarkers predictive values were subsequently calculated following retrospective disclosure of the infection classification data by the San Matteo and Maccabi lab investigators.

CMV DNA load analysis by RT-qPCR

DNA was extracted from 200 microliters of amniotic fluid samples yielding 50 microliters of eluted DNA, using MagnaPure (Roche Life Science, Indianapolis, IN). Viral DNA load (genome copies/ml) was determined by quantitative RT-PCR assay as previously described, with the use of primers and probes derived from the CMV glycoprotein B (for quantitation) and immediate early genes (28). The assay demonstrated a linear quantitation over a 6-log range with a sensitivity of 50 copies/ml.

Sample preparation, liquid chromatography-Mass Spectrometry, and data processing

Sample preparation: Amniotic fluid (150 μL) was concentrated to 50 μL using a 3 kDa MWCO filter (Amicon Ultracel-3K, Merck Millipore, Cork, Ireland). The concentrated fluid was loaded onto a serum depletion column (Multiple affinity removal column, 4.6x100 mm, Hu-14, Agilent) connected to a HPLC (1260 Infinity II Bio-Inert LC System, Agilent). After purging the system with buffers A and B, the depletion column was equilibrated in buffer A for 10 min at a flow rate of 1 mL/min at room temperature. The sample was loaded onto the column and the system was kept at 100% buffer A at a flow rate of 0.125 mL/min for 8 min, and then increased to 1 mL/min for another 2 min. The buffer was changed to 100% B at the same flow rate and kept for 18 min. The depleted fraction was collected at 5 to 15 min and underwent buffer exchange to 50 μL of 50 mM ammonium bicarbonate at a 1mg/ml concentration. Proteins were reduced with 5 mM dithiothreitol (Sigma) for 1 hr at room temperature, and alkylated with 10 mM iodoacetamide (Sigma) in the dark for 45 min at room temperature. Samples were diluted to 2 M urea with 50 mM ammonium bicarbonate. Proteins were then subjected to digestion with trypsin (Promega; Madison, WI, USA) overnight at 37°C at 50:1 protein:trypsin ratio, followed by a second trypsin digestion for 4 hr. The digestions were stopped by addition of trifluroacetic acid (1% final

concentration). Following digestion, peptides were desalted using Oasis HLB, µElution format (Waters, Milford, MA, USA). The samples were vacuum dried and stored in -80˚C until further analysis.

Liquid chromatography: ULC/MS grade solvents were used for all chromatographic steps. Each sample was loaded using split-less nano-Ultra Performance Liquid Chromatography (10 kpsi nanoAcquity; Waters, Milford, MA, USA). The mobile phase was: A) H2O + 0.1% formic acid and B) acetonitrile + 0.1% formic acid. Desalting of the samples was performed online using a reversed-phase Symmetry C18 trapping column (180 µm internal diameter, 20 mm length, 5 µm particle size; Waters). The peptides were then separated using a T3 HSS nano-column (75 µm internal diameter, 250 mm length, 1.8 µm particle size; Waters) at 0.35 µL/min. Peptides were eluted from the column into the mass spectrometer using the following gradient: 4% to 25%B in 155 min, 25% to 90%B in 5 min, maintained at 90% for 5 min and then back to initial conditions.

Mass Spectrometry: The nanoUPLC was coupled online through a nanoESI emitter (10 µm tip; New Objective; Woburn, MA, USA) to a quadrupole orbitrap mass spectrometer (Q Exactive HFX, Thermo Scientific) using a FlexIon nanospray apparatus (Proxeon). Data was acquired in data dependent acquisition (DDA) mode, using a Top10 method. MS1 resolution was set to 120,000 (at 400m/z), mass range of 375-1650 m/z, AGC of 3e6 and maximum injection time was set to 60 msec. MS2 resolution was set to 15,000, quadrupole isolation 1.7 m/z, AGC of 1e5, dynamic exclusion of 40 sec and maximum injection time of 60 msec.

Data processing: Raw data was processed with MaxQuant v1.6.0.16. The data was searched with the Andromeda search engine against the human and HCMV proteome databases appended with common lab protein contaminants and the following modifications: Carbamidomethylation of C as a fixed modification and oxidation of M, deamidation of N and Q and protein N-terminal acetylation as variable ones. MBR was

enabled and Label-Free Quantification (LFQ) min. ratio count was set to one. The rest of the parameters were kept as default. The LFQ intensities were extracted and used for further calculations using Perseus v1.6.0.7. Decoy hits were filtered out, as well as proteins that were identified on the basis of a modified peptide only and GO annotations added. The LFQ intensities were log transformed and only proteins that had at least three valid values in at least one experimental group were kept. The remaining missing values were randomly imputed. The t-test was performed to identify significant differential protein expression between the groups, and a p-value of 0.05 or less was considered statistically significant. To be defined as a differential protein expression we also required a minimal fold-change of > 1.5 between the groups.

The mass spectrometry proteomics data have been deposited to the ProteomeXchange Consortium via the PRIDE partner repository with the dataset identifier PXD029105.

A.

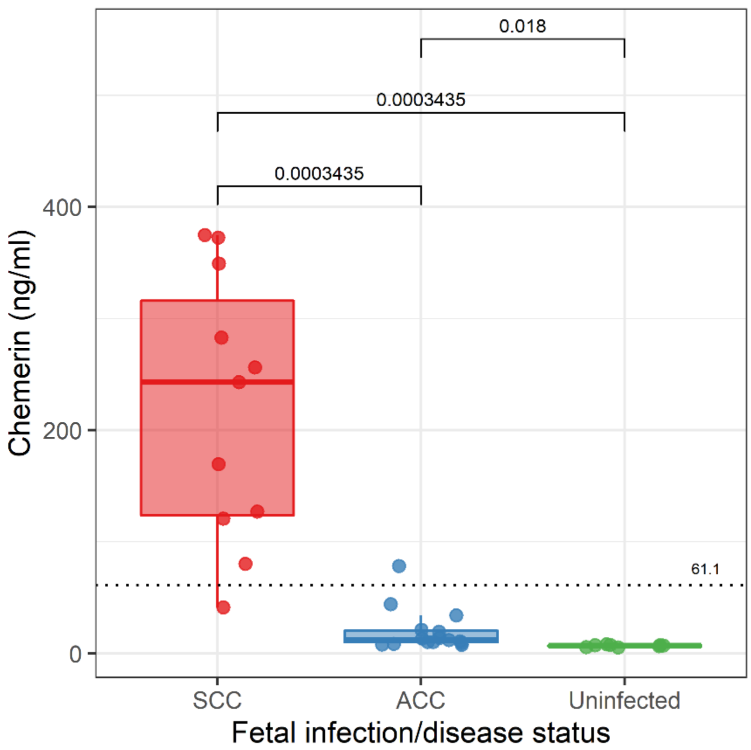
0.018
0.0003435
0.0003435
400
200
0
Chemerin (ng/ml)
61.1
SCC
ACC
Uninfected
Fetal infection/disease status

B.

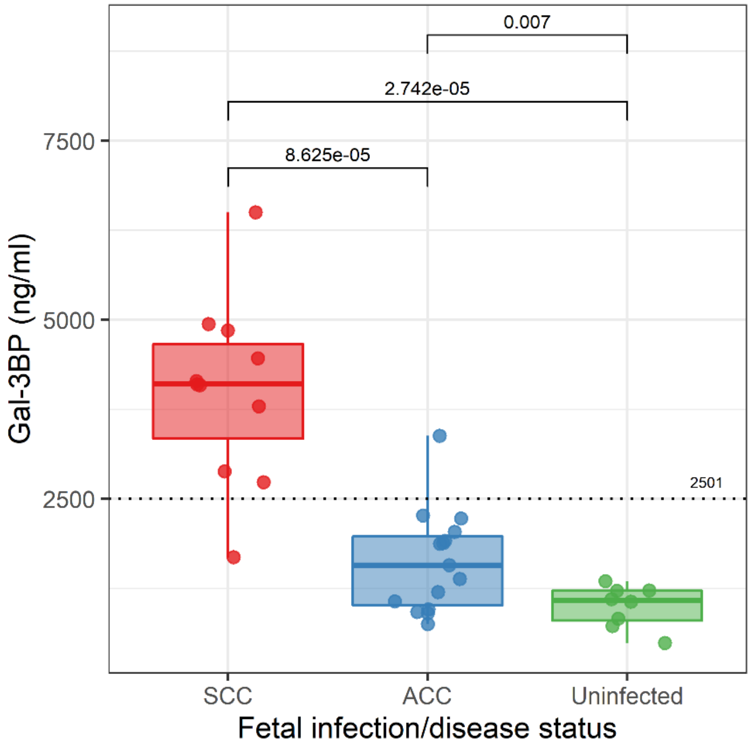
0.007
2.742e-05
8.625e-05
7500
5000
2500
Gal-3BP (ng/ml)
2501
SCC
ACC
Uninfected
Fetal infection/disease status

Figure S1. Amniotic fluid chemerin (A) and Gal-3BP (B) levels in an Initial Validation cohort of fetuses with severe cCMV (SCC; n=11), asymptomatic cCMV (ACC; n=15), and in uninfected fetuses (n=8).

The dotted horizontal lines represent optimal predictive cutoff values derived by ROC analysis. The figures were generated with the R ggplot2 package (version 3.1.0). Boxplots were generated with geom_boxplot default parameters, such that the box represents 25-75 percentile, and the whiskers from the box to the largest/smallest value no further than 1.5 * IQR from the box (where IQR is the inter-quartile range, or distance between the first and third quartiles). P-values were calculated by Bonferroni correction for multiple comparisons.

A.

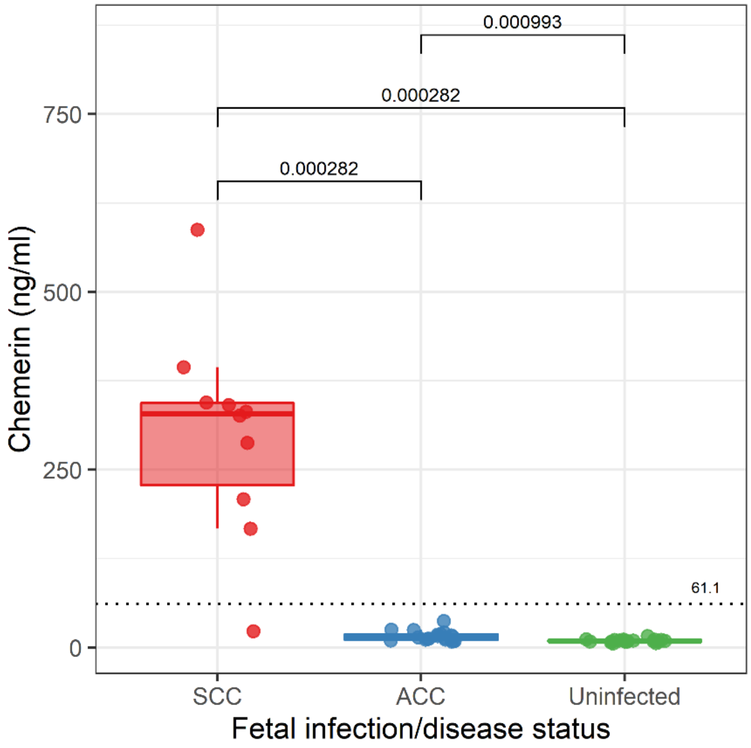

0.000993
0.000282
0.000282
750
500
250
0
61.1
Chemerin (ng/ml)
SCC
ACC
Uninfected
Fetal infection/disease status

B.

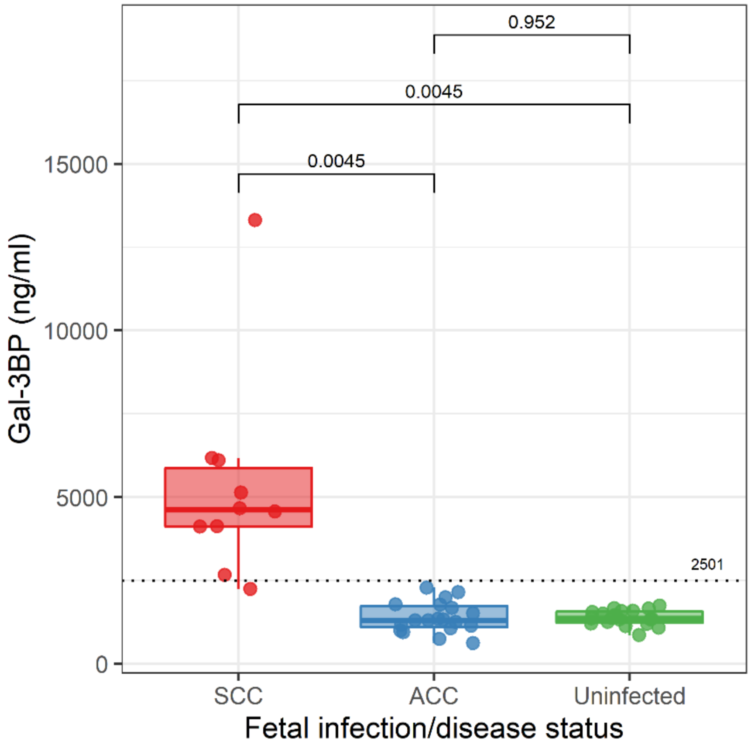

0.952
0.0045
0.0045
15000
10000
5000
0
2501
Gal-3BP (ng/ml)
SCC
ACC
Uninfected
Fetal infection/disease status

Figure S2. Amniotic fluid chemerin (A) and Gal-3BP (B) levels in a Blind-Testing cohort of fetuses with severe cCMV (SCC; n=10), asymptomatic cCMV (ACC; n=19), and in uninfected fetuses (n=19).

The dotted horizontal lines represent optimal predictive cutoff values derived by ROC analysis from an unrelated Initial Validation cohort. The figures were generated with the R ggplot2 package (version 3.1.0). Boxplots were generated with geom_boxplot default parameters, such that the box represents 25-75 percentile, and the whiskers from the box to the largest/smallest value no further than 1.5 * IQR from the box (where IQR is the inter-quartile range, or distance between the first and third quartiles). P-values were calculated by Bonferroni correction for multiple comparisons.

Table S1. Prenatal imaging data, outcome, infection severity classification, and amniotic fluid analyses in CMV-infected cases included in the Discovery cohort

Case no.	Imaging findings	Gestational age at amniocentesis (weeks)	Gestational age at appearance of severe imaging findings (weeks)	Pregnancy outcome	Postnatal follow-up (months)	cCMV severity classification	Proteome analysis	ELISA validation of specific biomarkers
98	US - hydrops (ascites, pericardial effusion, cardiomegaly)	21	22	TOP	NA	SCC	+	+
212	US - hyperecogenic ventricular wall, cortical and thalamic calcifications	21	22	TOP	NA	SCC	+	+
30	US - cortical calcifications, ventriculomegaly > 15mm, agenesis of corpus callosum, hyperecogenic bowel	23	27	TOP	NA	SCC	+	+
911	US - polymicrogyria, head circumference < 2 SD of normal; MRI - polymicrogyria, microcephaly, white matter cystic lesions	22	31	TOP	NA	SCC	+	+
302	US - temporal cysts; MRI - temporal cysts	22	33	TOP	NA	SCC	+	-
946	US – head circumference < 2 SD of normal, hyperechogenic bowel	22	22	TOP	NA	SCC	+	-
957	normal	22	NA	Delivery	>24	ACC	+	+
337	normal	22	NA	Delivery	>24	ACC	+	+
255	normal	23	NA	Delivery	>48	ACC	+	+
702	normal	22	NA	Delivery	>24	ACC	+	+
130	normal	21	NA	Delivery	>24	ACC	+	+
740	normal	23	NA	Delivery	>48	ACC	+	+
164	normal	23	NA	Delivery	>48	ACC	+	+
722	normal	23	NA	Delivery	>36	ACC	+	+

US = ultrasound; MRI = magnetic resonance imaging; SD = standard deviations; IUGR = intrauterine growth restriction; TOP = termination of pregnancy; NA = not applicable; cCMV = congenital cytomegalovirus; SCC = severe congenital CMV infection; ACC = asymptomatic congenital CMV infection

Table S2. Amniotic fluid proteins differentially-excreted between CMV-infected (n=14) and uninfected (n=10) fetuses

Protein	Gene Symbol	Protein ID (Accession number)	p value	Fold-Change
Epiphycan	EPYC	Q99645	<0.0001	9.1
Carbonic anhydrase 3	CA3	P07451	<0.01	7.1
Retinoic acid receptor responder 2 (Chemerin)	RARRES2	Q99969	<0.01	6.7
Signal-regulatory protein beta-1	SIRPB1	O00241	<0.0001	5.9
Bone marrow stromal antigen 2	BST2	Q10589	0.01	3.0
BPI fold-containing family B member 4	BPIFB4	P59827	0.02	2.9
Ubiquitin-like protein ISG15	ISG15	P05161	0.01	2.8
HLA class I histocompatibility antigen, Cw-2 alpha chain	HLA-C	P30501	<0.01	2.7
Glycoprotein nmb	GPNMB	Q14956	0.01	2.7
Keratin, type II cytoskeletal 6A	KRT6A	CON__P02538; P02538; CON__P50446	0.02	2.6
Dickkopf-related protein 1	DKK1	O94907	0.04	2.4
Fatty acid-binding protein, heart	FABP3	P05413	<0.01	2.4
ATP synthase subunit beta, mitochondrial	ATP5B	P06576	0.02	2.4
CD5 antigen-like	CD5L	O43866	0.04	2.4
Secreted and transmembrane protein 1	SECTM1	Q8WVN6	<0.0001	2.2
SH3 domain-binding glutamic acid-rich-like protein	SH3BGRL	O75368	<0.01	2.0
Carboxypeptidase Q	CPQ	Q9Y646	<0.01	2.0
Protein S100-A6	S100A6	P06703	0.01	2.0
Coagulation factor V; Coagulation factor V heavy chain; Coagulation factor V light chain	F5	P12259	0.02	2.0
Desmin	DES	P17661	0.02	2.0
Transmembrane emp24 domain-containing protein 7	TMED7	Q9Y3B3	0.03	2.0
Chitotriosidase-1	CHIT1	Q13231	0.03	2.0
Collagen alpha-1(IX) chain	COL9A1	P20849	0.02	1.9
Immunoglobulin J chain	IGJ	P01591	0.05	1.8
Galectin-3-binding-protein (Gal-3BP)	LGALS3BP	Q08380	0.03	1.7
Receptor-type tyrosine-protein phosphatase S	PTPRS	Q13332	0.02	1.7
Cellular retinoic acid-binding protein 2	CRABP2	P29373	0.04	1.7
Cathepsin B; Cathepsin B light chain; Cathepsin B heavy chain	CTSB	P07858	<0.0001	1.7
Laminin subunit alpha-5	LAMA5	O15230	0.02	1.6
Acidic leucine-rich nuclear phosphoprotein 32 family member A	ANP32A	P39687	0.04	1.6
Apoptosis-associated speck-like protein containing a CARD	PYCARD	Q9ULZ3	0.04	1.6
Low affinity immunoglobulin gamma Fc region receptor III-A	FCGR3A	P08637	0.04	1.6
Beta-2-microglobulin; Beta-2-microglobulin form pI 5.3	B2M	P61769	<0.0001	1.5
Cartilage acidic protein 1	CRTAC1	Q9NQ79	0.02	1.5

Pappalysin-2	PAPPA2	Q9BXP8	0.03	-1.5
WAP four-disulfide core domain protein 2	WFDC2	Q14508	0.05	-1.5
Collagen alpha-2(I) chain	COL1A2	P08123	0.02	-1.5
Vascular endothelial growth factor receptor 1	FLT1	P17948	0.03	-1.6
Lipolysis-stimulated lipoprotein receptor	LSR	Q86X29	0.01	-1.6
Kin of IRRE-like protein 1	KIRREL	Q96J84	0.05	-1.7
Collagen alpha-1(III) chain	COL3A1	P02461	0.01	-1.7
Palmitoleoyl-protein carboxylesterase NOTUM	NOTUM	Q6P988	0.03	-1.7
Insulin-like growth factor-binding protein 5	IGFBP5	P24593	0.01	-1.7
Isthmin-2	ISM2	Q6H9L7	0.03	-1.7
Glia-derived nexin	SERPINE2	P07093	0.03	-1.8
Protein HEG homolog 1	HEG1	Q9ULI3	0.04	-1.8
Bone marrow proteoglycan; Eosinophil granule major basic protein	PRG2	P13727	0.02	-1.8
Endogenous retrovirus group MER34 member 1 Env polyprotein	ERVMER34-1	Q9H9K5	0.02	-1.8
Aspartate aminotransferase, cytoplasmic	GOT1	P17174	0.02	-2.0
Amiloride-sensitive amine oxidase [copper-containing]	AOC1	P19801	0.02	-2.0
Serine protease 23	PRSS23	O95084	0.04	-2.1
Angiopoietin-related protein 6	ANGPTL6	Q8NI99	0.04	-2.1
Alpha-1,6-mannosylglycoprotein 6-beta-N-acetylglucosaminyltransferase A	MGAT5	Q09328	0.05	-2.2
Radixin	RDX	P35241	<0.01	-2.4
Protocadherin Fat 4	FAT4	Q6V0I7	<0.01	-2.6
N-acetylgalactosaminyltransferase 7	GALNT7	Q86SF2	0.01	-2.6
Immunoglobulin superfamily member 1	IGSF1	Q8N6C5	0.03	-2.6
Latrophilin-2	LPHN2	O95490	0.01	-2.7
Hepatocyte growth factor; Hepatocyte growth factor alpha chain; Hepatocyte growth factor beta chain	HGF	P14210	<0.01	-4.0

Included in the table are proteins differentially-excreted by > 1.5-fold
(-) before the fold-change value indicates decreased expression
The upper and lower bold horizontal lines mark proteins (shown above the upper line and below the lower line) differentially-excreted by > 2-fold

Table S3. Amniotic fluid proteins differentially-excreted between fetuses with severe (n=6) and asymptomatic (n=8) cCMV

Protein	Gene Symbol	Protein ID (Accession number)	p value	Fold-Change
Spectrin repeat containing nuclear envelope protein 1	SYNE1	Q8NF91	0.01	8.7
Sarboxypeptidase A2	CPA2	P48052	0.05	5.3
Retinoic acid receptor responder 2 (Chemerin)	RARRES2	Q99969	0.04	3.6
Glutathione S-transferase theta-1	GSTT1	P30711	0.04	3.0
C-reactive protein	CRP	P02741	0.05	2.5
Glycoprotein nmb	GPNMB	Q14956	0.03	2.5
Galectin-3-binding-protein	LGALS3BP	Q08380	0.02	2.4
Myosin heavy chain 14	MYH14	Q7Z406	0.03	2.1
Secretoglobin family 3A member 2	SCGB3A2	Q96PL1	0.06	2.1
Carboxypeptidase Q	CPQ	Q9Y646	0.05	1.7
Protein FAM3D (Family with sequence similarity 3 member D)	FAM3D	Q96BQ1	0.03	1.7
Keratin 17	KRT17	CON__Q04695; Q04695	0.04	1.7
Platelet derived growth factor receptor beta	PDGFRB	P09619	0.05	-1.5
ADAM metallopeptidase domain 9	ADAM9	Q13443	0.02	-1.5
Mesothelin; Megakaryocyte-potentiating factor; Mesothelin, cleaved form	MSLN	Q13421	0.05	-1.5
Sarcoglycan epsilon	SGCE	O43556	<0.01	-1.5
Growth arrest specific 1	GAS1	P54826	0.02	-1.6
Cell growth regulator with EF hand domain protein 1	CGREF1	Q99674	0.04	-1.6
Endoplasmic reticulum aminopeptidase 1	ERAP1	Q9NZ08	0.01	-1.9
Glycoprotein Ib platelet subunit alpha	GP1BA	P07359	0.02	-2.1
Actin alpha 1, skeletal muscle	ACTA1	P68133; P68032; P63267; P62736	0.05	-2.1
Alcohol dehydrogenase [NADP(+)]	AKR1A1	P14550	0.01	-2.5
Neurofascin	NFASC	O94856	0.04	-2.6
Chromogranin B	CHGB	P05060	0.03	-3.0
Pro-platelet basic protein	PPBP	P02775	0.01	-3.1
Lysosome-associated membrane glycoprotein 2	LAMP2	P13473	0.05	-3.3
Maltase-glucoamylase	MGAM	O43451	0.02	-3.6
Heat shock protein beta-1	HSPB1	P04792	0.04	-3.7
Angiopoietin like 6	ANGPTL6	Q8NI99	<0.01	-4.0

Included in the table are proteins differentially-excreted by > 1.5-fold
(-) before the fold-change value indicates decreased expression
The upper and lower bold horizontal lines mark proteins (shown above the upper line and below the lower line) differentially-excreted by > 2-fold

Table S4. Prenatal imaging data, outcome, infection severity classification, and amniotic fluid analyses in CMV-infected cases included in the Independent Validation cohort

Case no.	Imaging findings	Gestational age at amniocentesis (weeks)	Gestational age at appearance of severe imaging findings (weeks)	Pregnancy outcome	Postnatal follow-up (months)	cCMV severity classification	Proteome analysis	ELISA validation of specific biomarkers
407	US - periventricular hyperechogenicity and cysts, small cerebellum, hyperechogenic bowel	23	23	TOP	NA	SCC	-	+
181	US - periventricular hyperechogenicity and cysts, hyperechogenic bowel	21	23	TOP	NA	SCC	-	+
758	US - periventricular hyperechogenicity, intracerebral calcifications	27	23	TOP	NA	SCC	-	+
333	US - porencephalic cyst in the occipital lobe; MRI - occipital and temporal cysts	22	28	TOP	NA	SCC	-	+
614	US - periventricular calcifications, head circumference < 2 SD of normal, mild ventriculomegaly, hypoplasia of corpus callosum hyperecogenic bowel, liver calcifications	21	16	TOP	NA	SCC	-	+
254	US - periventricular calcifications, intraventricular adhesions	22	29	TOP	NA	SCC	-	+
486	US - intraventricular adhesions, temporal cysts; MRI - bilateral temporal cysts	22	31	TOP	NA	SCC	-	+
IT 1	US - periventricular hyperechogenicity, head circumference < 2 SD of normal	20	21	TOP	NA	SCC*	-	+
IT 2	US - periventricular hyperechogenicity, head circumference < 2 SD of normal	21	21	TOP	NA	SCC*	-	+
IT 3	US - head circumference < 2 SD of normal, periventricular hyperechogenicity, enlarged Cisterna Magna	21	21	TOP	NA	SCC*	-	+
IT 4	US - mild ventriculomegaly, periventricular hyperecogenicity, hyperecogenic bowel	21	21	TOP	NA	SCC*	-	+

IT 18	US - periventricular hyperechogenicity, corpus callosum hypoplasia, placentomegaly	21	21	TOP	NA	SCC*	-	+
IT 20	US – lissencephaly, head circumference < 2 SD of normal, IUGR	21	21	TOP	NA	SCC*	-	+
IT 21	US - periventricular hyperechogenicity, lissencephaly	21	21	TOP	NA	SCC*	-	+
IT 22	US - head circumference < 2 SD of normal, lissenecephaly, mild ventricolomegaly	20	20	TOP	NA	SCC*	-	+
IT 23	US - 21w: normal; MRI - 28w: polymicrogyria	21	28	TOP	NA	SCC*	-	+
IT 29	MRI - 25w: periventricular cystic lesions; MRI - 30w: intraventricular synechiae, periventricular pseudocystis	20	25	Delivery	>12	SCC*	-	+
431	normal	22	NA	Delivery	>48	ACC	-	+
873	normal	23	NA	Delivery	>36	ACC	-	+
408	normal	21	NA	Delivery	>24	ACC	-	+
902	normal	22	NA	Delivery	>24	ACC	-	+
472	normal	22	NA	Delivery	>36	ACC	-	+
943	normal	22	NA	Delivery	>24	ACC	-	+
859	normal	22	NA	Delivery	>18	ACC*	-	+
IT 5	normal	20	NA	Delivery	>48	ACC*	-	+
IT 6	normal	20	NA	Delivery	>36	ACC*	-	+
IT 7	normal	20	NA	Delivery	>36	ACC*	-	+
IT 8	normal	20	NA	Delivery	>36	ACC*	-	+
IT 9	normal	20	NA	Delivery	>24	ACC*	-	+
IT 15	normal	20	NA	Delivery	>48	ACC*	-	+
IT 16	normal	20	NA	Delivery	>48	ACC*	-	+

IT 17	normal	20	NA	Delivery	>36	ACC*	-	+
IT 19	normal	20	NA	Delivery	>12	ACC*	-	+
IT 27	normal	20	NA	Delivery	>12	ACC*	-	+
IT 28	normal	20	NA	Delivery	>12	ACC*	-	+
IT 30	normal	20	NA	Delivery	>24	ACC*	-	+
0739	normal	22	NA	Delivery	>36	ACC*	-	+
6914	normal	22	NA	Delivery	>36	ACC*	-	+
5886	normal	22	NA	Delivery	>36	ACC*	-	+
5081	normal	22	NA	Delivery	>36	ACC*	-	+
6423	normal	21	NA	Delivery	>24	ACC*	-	+
3408	normal	22	NA	Delivery	>24	ACC*	-	+
1481	normal	22	NA	Delivery	>18	ACC*	-	+

* Blind Testing

US = ultrasound; MRI = magnetic resonance imaging; SD = standard deviations; IUGR = intrauterine growth restriction; TOP = termination of pregnancy; NA = not applicable; cCMV = congenital cytomegalovirus; SCC = severe congenital CMV infection; ACC = asymptomatic congenital CMV infection

Table S5. AUC, sensitivity, specificity, and predictive values of chemerin and Gal-3BP amniotic fluid levels in relation to cCMV severity

			AUC [95% CI]	Cutoff values (ng/ml)*	Sensitivity (%)	Specificity (%)	PPV (%)	NPV (%)
**Initial Validation cohort**	SCC (n=11) vs. ACC (n=15)	Chemerin	0.988 [0.957-1.0]	61.1	90.9	93.3	90.9	93.3
		Gal-3BP	0.945 [0.855-1.0]	2501	90.9	93.3	90.9	93.3
**Blind Testing cohort**	SCC (n=10) vs. ACC (n=19)	Chemerin	NA	61.1	90.0	100	100	95.0
		Gal-3BP	NA	2501	90.0	100	100	95.0

*Defined by ROC analysis of the Initial Validation cohort
AUC = area under the curve; Gal-3BP = Galectin-3-binding-protein; cCMV = congenital cytomegalovirus; SCC = severe congenital CMV infection; ACC = asymptomatic congenital CMV infection; PPV = positive predictive value; NPV = negative predictive value
